# COVID-19 oriented HRM strategies influence on job and organizational performance through job-related attitudes

**DOI:** 10.1371/journal.pone.0266364

**Published:** 2022-04-13

**Authors:** Agnieszka Bieńkowska, Anna Koszela, Anna Sałamacha, Katarzyna Tworek

**Affiliations:** Faculty of Management, Wroclaw University of Science and Technology, Wroclaw, Poland; Satyawati College (Eve.), University of Delhi, INDIA

## Abstract

The COVID-19 crisis forced many changes to occur within organizations, which were necessary to keep the continuance of the organization’s operations. Job performance seems to be an important factor determining such continuance, through its influence on the performance of entire organization. Shaping and keeping job performance in times of COVID-19 pandemic was a challenge for organizations, due to its negative impact on employees, causing their stress or lack of sense of security. There is a growing role of HRM specialists in appropriately shaping HRM strategies that can positively shape job-related attitudes, resulting in enhanced job performance during such difficult times. Therefore, this study aims to explain the role of COVID-19 oriented HRM strategies in shaping job performance through job-related attitudes such as work motivation, job satisfaction, and organizational commitment in a time of crisis occurring in the organization due to the COVID-19 pandemic. The study was conducted among 378 organizations operating in Poland during 2^nd^ wave of COVID-19 pandemic. To verify the hypotheses, descriptive statistics were calculated using IBM SPSS and path analysis was performed using IBM AMOS. The result shows that combined set of "hard" HRM strategies related to the financial aspects and "soft" HRM strategies related to keeping employees’ wellbeing during the crisis gives the best results in shaping job performance through job-related attitudes and consequently strengthening organizational performance. This study contributes to the knowledge concerning the development of COVID-19 oriented HRM strategies, which may also have practical application.

## 1. Introduction

There is no doubt that employees are a key resource of contemporary organizations [1]. Employees, by implementing the tasks entrusted to them, support the implementation of the organization’s goals at the strategic, tactical and operational levels, which is translated into the performance of individual areas of the organization [2]. The global pandemic caused by SARS-CoV-2 virus (further called COVID-19 pandemic) had a profound impact on the management of employees [3, 4]. Spread of the COVID-19 disease meant that organizations were forced to change the way of work, as it became necessary to increase the social distance and stop the mobility in order to reduce the odds of contamination [5]. Organizations had to rearrange their processes so that remote work was possible [6]. Employees faced the challenges related to working from home: necessity to prepare the workplace, adaptation of tools to those that are possible to work from a distance, transformation of business interactions to the Internet and finding themselves in virtual reality [7]. This completely changed the perception of the work as well as the approach to performance.

It is worth to underline that the changes caused by the COVID-19 pandemic are not entirely revealed. Previous studies do not clearly indicate the impact of changes that took place in the organization on the results of employees’ work and, consequently, on the results of the organization as a whole. On the one hand, there is research that shows negative impact. Uncertainty of employment, increased level of stress, shortage of tools for work, technical problems, longing for the working environment, difficulties in building relationships with colleagues, disturbed work-life balance may adversely affect the results of employees [8–10]. Organizations also predict the negative effects of the pandemic—46% of surveyed Chinese companies expect a reduction in performance due to COVID-19 [11]. On the other hand, there has been much evidence of a positive impact. Graves & Karabayeva [9] have shown in their research that the effects of remote work such as flexibility of working time, no need to travel or the possibility of recruiting employees from anywhere in the world increase employee performance. Narayanamurthy & Tortorella [11] also agree with this claim, concluding that remote work contributes to increasing productivity, job satisfaction and output quality, and this translates into better job performance. Moreover, the authors noticed that those aspects easily translate into organizational performance. Bearing in mind the above suggestions and inconsistencies in the results of the research, the research gap emerged.

In this context, the aim of the article is to explain how COVID-19 oriented Human Resource Management (HRM) strategies influence organizational performance. In particular, the dependencies between the implementation of COVID-19 oriented HRM strategies, job performance and organizational performance will be examined. In addition, the mediating effects of job-related attitudes such as work motivation, job satisfaction and organizational commitment will be considered. The proposed and empirically verified model will allow the researchers to fill in the identified research gap.

## 2. Theoretical background

### 2.1 Job performance during COVID-19

The difficult conditions of the modern economy have placed organizational performance at the center of modern organizations’ attention [2, 12], in particular as a vital part of monitoring organizations’ progress [13]. It is also worth noting that in the field of management sciences, it has become a construct which is receiving a lot of attention [14–17]. Organizational performance is defined as the ability to acquire and transform various types of resources (including human, financial or technical) in order to carry out tasks and achieve the goals of the organization [18]. This construct covers diverse domains of organizational outcomes, such as: financial performance, product market performance and shareholder return, therefore requires synergistic involvement of entire organization. Consequently, individual areas of the organization (e.g. marketing, operations, strategy) are assessed by their contribution to organizational performance [12]. HRM seems to be considered as extremely important in shaping such performance [19]. Many authors emphasize the role of factors related to HRM in shaping organizational performance, in particular, the impact of job performance is considered [13, 20], usually as the crucial one [21].

Research performed by various authors, e.g. by Ramezan and colleagues [18] showed that there is a positive relation between job performance and organizational performance. The authors argue that various aspects of job performances can influence different aspects of an organization. Resource allocation decisions have a direct impact on employees’ productivity and organizational performance through reduced turnover, employees’ best behavior and the overall task performance of employees. All the above-mentioned aspects consequently influence organizational performance [18]. This is supported by Bieńkowska et al. [22], who emphasize that thanks to highly performing employees, an organization can carry out tasks and achieve goals much more efficiently [22]. Additionally, Teece [19] claims that the organization’s ability to create, implement, protect and use intangible knowledge assets allows achieving superior organizational performance [23].

It is equally common knowledge that modern organizations face the challenges of operating in the changing environment on a daily basis. Masa’deh et al. [24] argue that the job performance of individuals should be placed at the center of every organization’s interest, so they would be able to ensure business continuity and succeed in difficult conditions [24]. This was especially true during the COVID-19 pandemic. The crisis, that the whole world had to face, increased the uncertainty of the organization’s operation, and thus the turbulence of the environment. Employees’ behavior, manifested in job performance, supports organizations in resisting the negative consequences of the pandemic [25]. It is worth noting, however, that there are many reports that the COVID-19 pandemic decrease job performance.

One aspect caused by the spread of contamination is the need for social isolation. In quantitative research on salesperson Chaker et colleagues [26] found that there is indirect impact social isolation on job performance. They indicated that job-related knowledge, informal communications, and loyalty to the organization are mediators in this relation. In addition, the qualitative research confirmed the assumed impact, showing the significance of hindered development and learning, missed opportunities for informal conversations, and detached feelings from the organization [26].

Another negative consequence of the COVID-19 pandemic was layoffs in certain sectors of the economy, including the hospitality industry [27]. Tu et colleagues [10] found that layoffs due to the COVID-19 increase the level of stress among employees who remain in the organization, which translates into decrease their in—role and extra—role performance [10].

Also noteworthy are the results of the Shin et colleagues [28] study, who conducted a comparative analysis of the impact of customer incivility behavior on the service employees performance from the pre-pandemic and SARS-CoV-2 virus pandemic period. It was discovered that there is stronger, indirect effect of customer incivility on job performance through emotional exhaustion during pandemic [28].

On the other hand Vo-Thanh et colleagues [25] noted the important role of organization in improving job performance. Research results showed that there is a positive relation between satisfaction with organization COVID-19 responses and job perfomance. The authors explain that when employees are satisfied with the organization’s anti-crisis activities, they are more confident about their wellbeing, health, and job, and this translates into better results [25]. That is why it is important to analyze and identify any ways, in which the organization can support the employees in maintaining the proper level of job performance, or even increase it–as it should be considered as a main factor enabling the organizational survival.

### 2.2 COVID-19 oriented HRM strategies

Unexpected events causing a global scale crisis can have a dramatic impact on the employees’ wellbeing [29, 30]. The situation caused by the COVID-19 pandemic is guaranteed to take a long-term effect on the psychological wellbeing of employees, causing cumulative stress for many of them, which will consequently affect performance, a key aspect for organizations.

Because employees are the most important resource of any organization, the organization needs to take care of their wellbeing as well [31, 32]. Therefore, a health disaster such as a pandemic will pose a huge challenge to the HRM department to mitigate the negative effects of the COVID-19 pandemic on employees [29, 33]. As early as the 2008 crisis, there were voices from researchers arguing that HR professionals should be part of the crisis management team.

During a COVID-19 pandemic, employees often face problems not only at work but also at home. Therefore, HR specialists should be ready to manage any problems and issues caused by the COVID-19 pandemic [29]. The HR department gains importance in the organization and its main task during a pandemic is to mitigate the negative effects of a global pandemic among employees [29]. Therefore, it is necessary to involve the HR department in the planning and implementation of crisis management as the main representative of the highest capital of any organization [31].

Researchers have noted that modern approaches to HRM are more effective in turbulent times than traditional approaches that are considered edgy and unprecedented [34]. Therefore, it is important to recognize which HRM practices will most effectively mitigate the negative effects of the COVID-19 pandemic, and also allow the use of emerging opportunities [29]. In turbulent times, two HRM approaches are distinguished from an organization’s perspective " soft"—in which employees are considered a valuable resource for the organization [35] and "hard". When considering "soft" HRM practices, they mainly focus on taking care of the employee’s wellbeing during the crisis to guarantee high performance.

Therefore, organizations focus on training employees to acquire new skills necessary to work in new conditions [36]. Additionally, they focus on appropriate communication processes, transfer and exchange of information [37, 38]. They also try to provide activities such as mentoring, coaching, which positively, even in crisis situations, raise the wellbeing of employees [39].

The most common „hard” HRM practices include those related to the financial aspect—pay cuts and freezes [40], downsizing [41, 42] or withholding recruitment, reducing training budgets, lowering individual performance goals and benefits [43, 44].

In the literature, mixing of "soft" HRM style with "hard" HRM style seems to be the most effective way to mitigate the negative impact of the COVID-19 pandemic on the organization’s employees. The mixed model allows an organization to create maximum employee job performance and efficiency by taking care of employee motivation and engagement using "soft" HRM practices. On the other hand, it allows the organization to persevere in the difficult time of crisis by cutting operational costs related to recruitment or employees’ development and promotions, thus ensuring its high efficiency [45].

Considering all that and the importance of job performance maintenance during COVID-19 pandemic (to ensure organizational performance), it seems that there is an apparent need to indicate what detailed HRM strategies will be beneficial for the organizations operating in such conditions in order to maintain and boost the job performance. The above-mentioned detailed HRM strategies should relate to three areas: securing qualified staff, adapting the organization to the new reality, and ensuring the wellbeing of stakeholders. Hiring and employee development must be taken into account as part of the provision of qualified personnel. In addition, it is necessary to discuss the strategy of adapting the work organization to changes that have occurred in the environment. It is necessary to take into account the need to move to virtual reality (digitalization), change the existing processes organizing work (job redesing), equipping employees with tools allowing to adapt to these changes (COVID-19 training). It seems obvious that the above issues will not be possible without ensuring a good flow of information (communication). Moreover, in such difficult circumstances, the issues of providing support to entities within and outside the organization (employee wellbeing and corporate social responsibility) should be taken into account.

#### 2.2.1 Hiring

COVID-19 pandemic has significantly interrupted an organization’s operations, and the need to create an action plan in response to a rapid and unexpected event is riddled with extreme uncertainty. Among other things, it is very difficult to predict the financial impact of the COVID-19 pandemic. Thus, it is difficult to assess the severity of the pandemic on the organization’s finances. There are many strategies that an organization adopts to protect the financial aspects of the organization by choosing to freeze certain expenses, including freezing dedicated hiring expenses [46]. The employees’ staffing includes two basic HRM activities: recruitment and selection [47]. All activities aimed at recruiting employees reflect the general mood of the business and the opportunities the organization will have once the pandemic is over [47].

Unfortunately, due to its high instability, future organizations are not able to predict their staffing needs enough even in the short term [47]. The dynamics of the staffing needs depend on a large number of factors, such as market demand or political regulations [47]. It is therefore the responsibility of the HR department to adapt to current trends.

Therefore, it is recommended to be very careful during analyzing not only future needs of resources but also current one [47]. Many sources demonstrate that the most commonly adopted strategy is that of freezing the recruitment of new employees, either partially or completely [46, 48]. Data obtained from the U.S. LinkUp portal in the shows that even with the onset of the spread of the coronavirus, there was an uncharacteristic drop in job advertisements for the period. More representative data will emerge in May 2020, where a 40% drop below the average level of ads for the same week in 2017–2019 was recorded [49]. That is due to the fact, that organizations as a result of the crisis predict a reduction in aggregate demand, and a reduction in employment or a complete freeze is a kind of response to the decline in demand [46, 50].

In a situation of constantly changing socio-economic conditions, such actions are primarily aimed at protecting current employees and reducing disruption to the organization [46].

#### 2.2.2 Employee development

Employees as a key component of any organization significantly affect its productivity, success, and future. This is the main reason why organizations decide to invest significant amounts in employee development [51]. In practice, employees’ development means not only developing their skills but also the organization as a whole. More productive employees also mean that the organization will also prosper better [52]. Although employees’ development and promotions are considered one of the most effective ways to support employees’ performance. In times of crisis, when the need to survive in the market turns out to be crucial, organizations decide to take many radical steps that eliminate the flexibility of the organization [53]. The HR department also has a key role in dealing with the consequences of the crisis, which is responsible for reducing stress among the employees in the organization, still taking care to invest in human capital, but keeping in mind the minimization of strategic costs [54]. As it was mentioned, during a crisis, organizations very often decide to reduce many of the additional costs, which also result from the payment of additional benefits, promotions, and employees’ development [54]. Therefore, a crisis away opportunities related to employees’ development and promotions, despite rising employee expectations. Moreover, as has already been mentioned, organizations are also often forced to reduce or completely cut down on recruitment, therefore HR departments have to prepare tools that will smooth the situation and keep current employees in the organization, despite the growing risk of employee dissatisfaction associated with the need to reduce promotions and employees’ development [54].

Among these tools are modern methods of offering alternative jobs, part-time positions, or flexible working hours [55]. This provides employees with a certain sense of security, which in times of global crisis will have a more effective impact on employees, their performance, and their intention to stay in the organization. Thus, issues related to development and promotion opportunities will come to the background, so that their reduction during the crisis will not have a significant impact on employees and their performance [56].

#### 2.2.3 Digitalization

Despite the many often drastic changes needed to be made in an organization under COVID-19, there are some forms of restructuring, which can help overcome the negative effects of the COVID-19 pandemic [57]. Indeed, recently there has been an increased digitalization in organizations, the application of which often overcomes or significantly reduces the negative effects of the pandemic.

Organizations in a very short time have been forced to move their products online or to implement more information systems necessary for the continued functioning of the organization [57]. Digitalization is characterized by the combination of certain advanced technologies and the integration of physical and digital systems [58]. Digitalization is a process of transformation occurring in an organization through the adoption of digital technology [59–61] manifested as digital artifacts or digital platforms [62–66], but also in the form of a digital business model and management, including human resources [67].

Digital technology is considered as a potential that implemented in an organization on a large scale, will gain competitive advantage by improving organizational flexibility and resilience [66], as well as increasing dynamic capabilities. Therefore, digitization will be beneficial for dynamic changes in the organization, also caused by the dynamic environmental changes, because can helps to recognize and sense changes in the environment [60, 68, 69]. During the pandemic, it was noted how important digitization is in an organization.

The implementation of digitalization has helped to create many new opportunities and thus allowed organizations to survive the difficult time of the pandemic and provided new opportunities for growth [70]. Digitalization has identified innovative opportunities to utilize new technologies like the reconfiguration of an organization’s resources that effectively allows it to respond to crises [59]. Therefore, many organizations to maintain continuous growth and ensure the organization’s sustainability have also reconfigured HRM processes such as recruiting or onboarding new employees to ensure the safety of new and existing employees while providing the organization with adequate resources.

#### 2.2.4 Job redesign

Job design plays an important role in HRM processes because it considers, not only all structural and social aspects but also the workplace’s impact on the employees [71]. Furthermore, once a job design is made, it must be systematically reviewed and redesigned because many factors such as management style, working conditions, the technology used in the organization, and environmental dynamics influence how the job will look [71].

The COVID-19 pandemic has caused some changes in job design on organizations’ global level [72]. HRM professionals in organizations must ensure that they align the organization’s operations, and employees, with the changes. They also should support employees in adapting to these changes to ensure long-term benefits in improving job redesign [72].

Many organizations during the COVID-19, to avoid business downtime, quickly had to work under changed conditions: using new technologies, in entirely new spaces—most often at home or in hybrid mode, with reduced social and physical interaction, and with far less supervision and support from other colleagues [72]. Changes like work are not just about adapting to new technologies or automation, they often reflect changes in the way tasks are conceptualized and performed, such as treating patients online or providing customer service to restaurants online [73].

This is why job redesigning plays such a key role in the functioning of an organization. The current pandemic indicates that the changes implemented will persist for a long time and even remain in the organization even after the pandemic is over. Therefore, it is important for HRM professionals, to conduct a proactive review of job design, risk analysis, regulations, guidelines, and practices to protect the employees’ health in the future [72].

#### 2.2.5 COVID-19 Training

For many organizations, technical aspects, were not foreign even before the pandemic, due to the rapid development of automation and information technology. However, a much lower need for technology was observed before pandemic, especially among small organizations. The pandemic COVID-19 and the need to work from home has made pressure to implement technology at every organizational level. Therefore, a significant increase in the use of technology is being seen in all organizations, especially the small ones [74]. Such changes in the organization and working conditions, the increased need to use technology forced by the global COVID-19 pandemic causes stress, uncertainty and bitterness which can affect the employee’s work negatively [47].

The additional training organizing by the organization, providing support for employees in shaping new skills to help during this new mode of work and dealing with the new situation, could be very helpful. It would reduce employees’ stress and additionally give them a sense of development and acquisition of new knowledge and skills [75]. Accordingly, training that develops remote work skills will play an important role and organizations will need to quickly adapt their training policies to meet current requirements [74]. Moreover, for all organizations especially which operate in a global marketplace, seems to be useful to provide training about how to create a strong international culture and employees’ relationships online to ensure high employee engagement and wellbeing.

The ability to work remotely is becoming an increasingly desirable skill in the labor market, not only among managers but also among technical workers [76]. Therefore, these skills should be supported by organizing additional training that covering not only technical aspects but also experiential aspects of virtual work [75, 77, 78].

#### 2.2.6 Communication

An organization is considered a complex network of communication habits. However, the organization itself is only a structure, while the communication behaviors of employees build the organization as a whole [79, 80]. It is considered in this way because the employees exchange information about the organization, the work, and the goals being achieved [81, 82]. Communication in an organization takes different shapes depending on what type of messages employees are operating.

Communications can be vertical and horizontal, which means that communication will then be formal or informal [82, 83]. Communication itself can also be differentiated according to the function it is supposed to perform, namely: directive communication, aimed at influencing the receiver [81, 84, 85], supportive communication aimed at conveying relevant and reassuring information [83, 86, 87] cultural communication aimed at providing the internal rules of the organization [88, 89] and democratic communication involving employees in decision-making processes.

Indeed, communication is a crucial element in managing human resources by motivating them and keeping them engaged and committed to the organization [90]. Especially during the dynamic changes caused by the COVID-19 pandemic, appropriate communication will influence employees’ emotional commitment to organizational change, enhance their trust in the organization, and stimulate their commitment to the organization [90–94]. Researchers indicate that informal communication can reduce employees’ resistance to developing new skills and building new roles resulting from restructuring during COVID-19 [95, 96]. Moreover, keeping employees informed about important aspects of the pandemic or the future of the organization allows them to engage themselves in the continued development of the organization, reduces feelings of discomfort, uncertainty, and other negative emotions that can disrupt feelings of job satisfaction or job performance [97–99].

#### 2.2.7 Wellbeing

The radical changes occurring during the COVID-19 pandemic and the need to fulfill the obligation of social isolation and also the financial uncertainty causing by the successive closure of other and other sectors by the states have a very strong impact on the physical as well as mental health of people [100]. All kinds of difficult situations affecting societies, cause a direct negative impact on people’s mental and physical health, as proven by the research conducted in China during the COVID-19 pandemic, confirming high rates of stress and depression among people [101–104].

From the organizational point of view, stress, anxiety, or depression among employees, even if not directly related to their daily work, negatively affect their engagement, willingness to work, and consequently reduce their effectiveness [105, 106]. Organizations to help employees survive the difficult time of pandemics should especially focus on employee wellbeing.

The definition of wellbeing is not clear, as researchers view wellbeing as the ability to achieve goals [107]; happiness [108], and life satisfaction [109, 110]. On the other hand, Shin and Johnson [111] considered wellbeing in terms of a global assessment of a person’s quality of life according to their chosen assessment criteria and this definition is also relevant today [112–114].

In essence, stable wellbeing is when an employee has the psychological, social, and physical resources necessary to meet specific psychological, social, and/or physical challenges [110]. The COVID-19 pandemic has significantly affected the psychological balance of workers, preventing them from at least realizing the need for social exchange, the reduction of which will affect the sense of isolation in the workplace, thus resulting in stress [115]. What employers in particular need to take care of is free, unrestricted access to technology, other workers, and more free communication with employees, imitating a sense of face-to-face communication. Those actions can help in avoiding the risks associated with remote working [116]. Therefore, the wellbeing of employees during a pandemic, especially those working remotely, will depend largely on interpersonal interactions and social aspects, which are particularly difficult to provide in this mode of work [115].

#### 2.2.8 Corporate social responsibility

Many definitions of Corporate Social Responsibility (CSR) and organizational sustainability have emerged over the years, and the diversity of these definitions has big consisted of a focus on other stakeholder groups [117, 118]. One of the oldest definition present CSR as the responsibility of organizations to pursue such strategies and make such decisions that are desired and valued by society [119, 120].

Today, CSR pays attention to the fact that business and society are related in ways that go across the critical relationship between employees, customers, suppliers, and the community [117, 121]. CSR is therefore considered a multidimensional construct. Its mechanisms, processes, and evolutions are driven by a set of complex external and internal, socio-cultural, and economic factors, making it a dynamic, constantly evolving practice [122].

Therefore, its contemporary definition presents CSR as a discretionary allocation of corporate resources to improve social wellbeing, serving as a way to strengthen relationships with key stakeholders [123]. This theory indicates how much alignment is needed between stakeholder expectations and social wellbeing [124].

In particular during a crisis, an organization has responsibilities and obligations to its stakeholders. Thus, there is a requirement for organizations to support society and their important stakeholders, namely employees and customers, by engaging in CSR activities. To meet these demands, many organizations have adopted CSR as a form of a completely new global governance that promotes the ability to make collective decisions on transnational issues [125].

In a way, some kind of an examination has been forced on CSR organizations by the COVID-19 pandemic, which verifies a commitment to ethical business conduct [125, 126]. Indeed, during the pandemic, organizations have taken special responsibility for the safety of their employees. They try to provide for employees working in the home office the conditions similar to the conditions in the office, but for those who cannot take advantage of remote work, they provide maximum security in the workplace and full security equipment.

Organizations somehow felt the need to take care of the community by promoting public health, providing medical assistance, financial support, or helping other sectors that were particularly affected by the negative effects of the pandemic. This is because such actions bring a sense of stability and strengthen the organization’s community [127].

## 3. Hypotheses development

### 3.1 HRM strategies influence job and organizational performance through motivation

In the context of the theoretical background described above, there seems to be a need to clarify the mechanism of HRM strategies influence on job performance while analyzing the mediating role of work motivation.

The truth is that properly selected HRM strategies themselves strengthen employees’ job performance [128]. However, given that work motivation, defined as the level of employee motivation to perform work effectively [129], can be stimulated by external factors such as the organization’s environment and culture (including HRM practices) it is assumed that it is necessary is to include work motivation in the consideration.

HRM strategies are composed of many practices that help an organization effectively manage employees from selection to exit [130, 131]. HRM as a modern approach to personal management, characterized by a closer alignment with the business, involves line management to care of aspects such as organizational commitment and work motivation. Properly selected HRM practices are a kind of cure for motivating employees in terms of knowledge, attitudes, and way of acting.

Taking additional actions to give the employee a sense of security and satisfaction, such as: taking care of their comfort in the workplace or working from home, taking care of the wellbeing, their relationship with employees, appropriate use of new technologies, and properly adapted communication process will compensate anxiety caused by COVID-19 pandemic and contribute to increasing their work motivation [132, 133]. In turn, employees’ positive work perceptions may motivate them to invest maximum effort in their work to achieve their personal goals, thereby maximizing their job performance [132, 134]. The researchers recognized that HRM strategies directly affect employee motivation, behavior, and skill enhancement to increase their performance and increase organizational performance [130]. Therefore, taking care of the employees’ work motivation, as a result, provides a high level of employee performance with high-cost effectiveness through the full exploitation of employees’ potential [128].

Taking into account the above consideration, it is necessary to formulate a hypothesis:

*H1*: *COVID-19 oriented HRM strategies influence job performance through work motivation*.

### 3.2 HRM strategies influence job and organizational performance through satisfaction

Similar to the case of work motivation considered as a construct that strengthens the effect of HRM strategies on job performance, it seems important to verify the mechanism of the effect of HRM strategies on job performance, while including the mediating role of job satisfaction.

Job satisfaction is the result of many work-related experiences, both positive and negative. For employees, it represents the level of satisfaction and positive emotions towards the job [135, 136].

Increasing stress among employees or a sense of uncertainty caused by the COVID-19 pandemic makes it significantly more difficult to positive strengthen job satisfaction [137]. Therefore, it seems to be especially important to choose the right HRM practices that will support the employee and provide a sense of fulfillment of their needs. HRM practices try to develop and locate human capital in such a way as to achieve long-term goals, providing them with multiple benefits, thus stimulating job satisfaction [138]. It is repeatedly indicated that job satisfaction consists of factors such as relationship with superiors pay, opportunities for advancement and development, and relationship with co-workers, all aspects that are somewhat shaped by HRM [139].

The fulfillment of the employees’ needs, in turn, provides incentives that can motivate them to optimize their daily work and increase their job performance [135]. Many researchers confirm the influence of HRM practices on job satisfaction and the positive impact of these factors on employees’ job performance [140, 141].

It has been proven several times that job-satisfied employees are also high-performing employees [135, 142]. Employees who have positive perceptions about the organization and their work tend to be more engaged in their tasks thereby exhibiting higher performance [143]. Therefore, maintaining a high level of job satisfaction among employees is critical to achieving employee quality and performance [137, 144, 145].

Taking into account the above consideration, it is necessary to formulate a hypothesis:

*H2*: *COVID-19 oriented HRM strategies influence job performance through job satisfaction*.

### 3.3 HRM strategies influence job and organizational performance through organizational commitment

It also seems important to verify the mechanism of influence of HRM strategies on job performance, while including the mediating role of organizational commitment.

Organizational commitment reflects employees’ positive sense of belonging to the organization. According to Meyer and Allen [146], organizational commitment is considered in the context of an employees’ emotional relationship with the organization, consisting of identification with it, a sense of responsibility for the organization’s success, a sense of fulfilling responsibilities to the organization, and avoiding the costs of leaving the organization [146, 147].

This construct is most often considered in three categories: affective commitment, continuance commitment, and normative commitment. Affective commitment refers to an employee’s involvement in social relationships inside the organization. Continuance commitment refers to an employee’s commitment to their social roles and their social position within the organization. Normative commitment focuses on the moral sense of obligation to stay in the organization, so employees decide to stay in the organization because of their values [146].

Reviewing the literature, one can find studies that support an increase in organizational commitment of employees through the use of appropriate HRM practices [148, 149]. An increase in organizational commitment is expected primarily through the use of practices that increase employee trust in the organization and provide a sense of security, which is especially important in times of crisis [150].

Taking care to: effectively communicate, engaging in CSR activities, supporting the employees during difficult times, and caring for their wellbeing, gives the employees a sense that they are a key part of the organization [151]. Therefore, a positive perception of management’s intentions by employees strengthens the employees’ organizational commitment [152].

Organizational commitment is rooted in the desire to satisfy internal needs in the workplace, which significantly motivates employees to work [153]. The internal energy triggered by high levels of organizational commitment results in positive employee behavior toward the organization focused on achieving organizational goals [154, 155].

Consequently, organizational commitment results in the quality of employees’ work [156], which can be closely related to employees’ job performance. Nevertheless, the direct positive effect of organizational commitment on job performance has also been repeatedly indicated by researchers [157–163].

Taking into account the above consideration, it is necessary to formulate a hypothesis:

*H3*: *COVID-19 oriented HRM strategies influence job performance through organizational commitment*.

### 3.4 Final model of hypotheses

Based on the above considerations, it seems important to validate a model that includes all the above considered job-related attitudes influencing the relationship between HRM strategies on the job and organizational performance. Therefore, this means that including into the model the three job-related attitudes that are relevant to the HRM strategies on the job and organizational performance relationship will be of significantly enhanced value in building job and organizational performance.

In the COVID-19 crisis, it seems that focusing on job-related attitudes such as work motivation, job satisfaction and organizational commitment at the same time will be very beneficial in building employees’ job performance and thus organizational performance. There is no doubt that well-chosen HRM strategies will enhance job and organizational performance through particular job-related attitudes. Considering employees’ comfort and relationships and supporting the employees in developing to the appropriate use of technology will enhance the employees’ motivation to perform tasks effectively [132, 134]. Ensuring the employees’ positive perception towards the organization and reducing stress at work will positively affect their job satisfaction [138, 143, 148]. On the other hand, the use of HRM strategies that will positively influence the employees’ trust towards the organization and enhance the desire to belong to this work environment, will be de facto stimulate their organizational commitment [148, 149].

Thus, researchers confirm that, in general, there is a set of HRM strategies that support job and organizational performance through work motivation, job satisfaction, or organizational commitment. Therefore, it seems important to verify whether the same HRM strategies were properly chosen to be mitigating the negative impact of the COVID-19 crisis on organizations, enhance job and organizational performance through both work motivation, job satisfaction, and organizational commitment in parallel.

Based on the above considerations, it is necessary to formulate a hypothesis:

*H4*: *COVID-19 oriented HRM strategies influence job performance through job-related attitudes*: *work motivation*, *job satisfaction and organizational commitment*.

**Fig 1:**
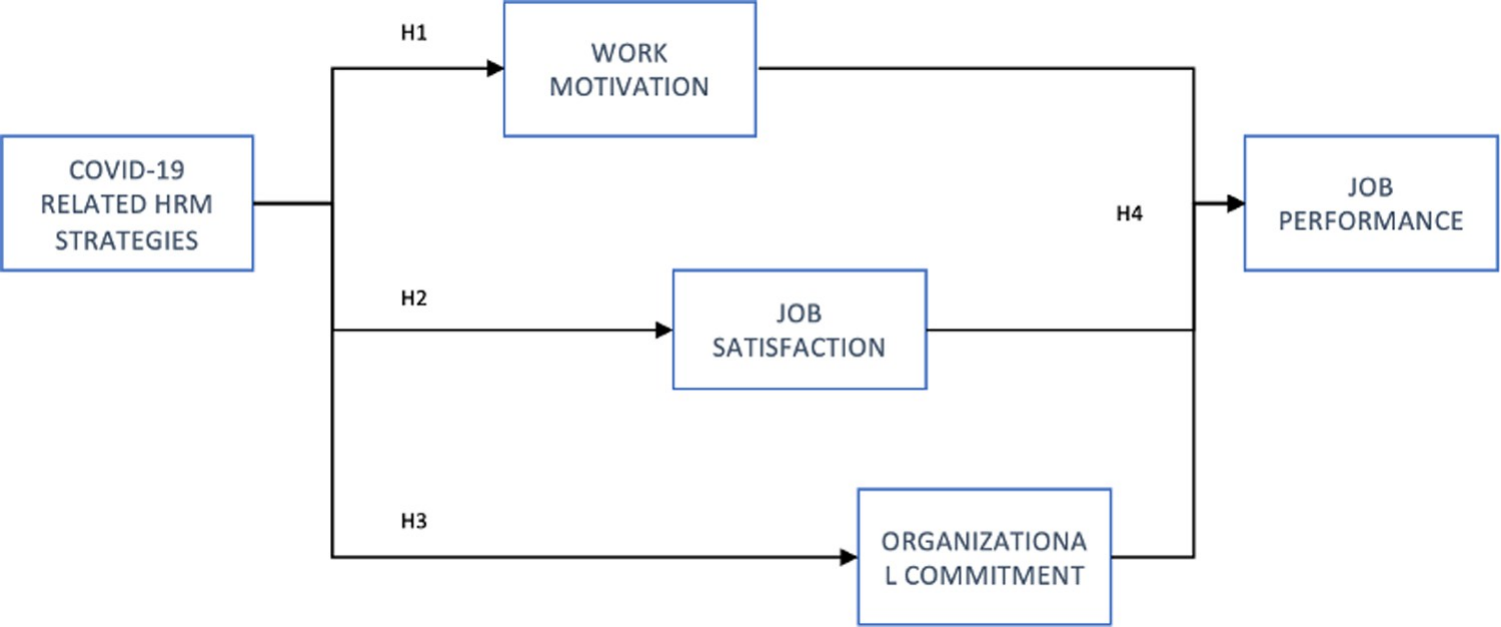
Hypotheses overview. Source: own work.

The overview of hypotheses is presented in Fig 1. The hypotheses will be verified using empirical research performed in a way, which allows to measure each variable and perform statistical reasoning allowing for identification of the relations between them.

## 4. Research methodology

### 4.1 The process of collecting data and the characteristics of the research sample

In order to achieve the goal of the article as well as to verify the hypotheses, a questionnaire study was conducted. The survey took place in the last quarter of 2020. There are two stages in which the research was carried out. The first stage is the pilot study aimed at determining the quality of the research tool. The pilot studies are treated as a prerequisite for using the questionnaire as a reliable research method [164]. They were conducted among 25 managers who played the role of competent judges. Their comments made it possible to improve the research tool used in the second stage of research and avoid any common method bias. The second, main stage of the research covered 378 organizations operating in Poland. The study was conducted using the CAWI (Computer-Assisted Web Interview) method. The selection of the elements for the sample was purposive, the only limitation was the geographical scope of the activity. The non-response bias was eliminated by the use of professional respondents’ panel. Other characteristics of the organization—for example the size of the organization or the main source of income did not affect the decision to include it in the research sample, but they were also examined. Detailed information on the surveyed organizations are presented in Table 1. Despite the fact that the selection of elements for the sample is not representative, it seems possible to formulate conclusions due to the diversity of the organization.

**Table 1:** Research sample characteristic.

Criterion of division	manufacturing organisations	trade organisations	service organisations	Total
micro (below 10 employees)	10	12	8	30
small (10–49 employees)	29	56	33	118
medium (50–249 employees)	45	27	43	115
large (above 249 employees)	46	14	55	115
Total	130	109	139	378

Source: own work.

### 4.2 Variables characteristic

To allow verification of hypotheses the following variables were used: Hiring, Employee Development, Digitalization, Job Redesign, COVID-19 Training, Communication, Wellbeing, Corporate Social Responsibility, Organizational Performance, Job Performance, Motivation, Satisfaction, Organizational Commitment (Table 2).

**Table 2:** Summary of the items and the corresponding variables.

Variable	Item
Hiring *HiringC	Due to COVID19 recruitment and hiring processes has been cancelled or limited for key/critical roles
Employee Development *EmpDevC	Due to COVID-19 development and promotion programs offered to employees have been suspended or limited to a minimum
Digitalization *DigitC	Organization applied virtual platforms for their recruitment and on boarding activities
Job Redesign *ReDesiC	Organization offered alternative work location (work-from-home, hybrid or rotation mode) and flexible work hour arrangements
	Organization created new skill demands, especially those related to the adoption of information and communication technology
COVID-19 training *TrainC	Organization provided support to shape new skills in connection with a new mode of work / situation by trainings and other forms
Communication *CommuC	Employees stayed informed of recent developments regarding health and safety, and self-development and training opportunities that can help employees adapt themselves to changing roles and requirements
Wellbeing *WellbeC	Organization developed new training topics included COVID-19 safety training, coping with stress and anxiety, and lifestyle-based training
	Organization had formally appointed a counselor to help people cope with mental health issues
	Organization provided work-from-home employees with laptops, printers and scanners etc.
Corporate Social Responsibility *CSRC	Organization has been extensively engaging in CSR activities to support underprivileged people during COVID-19
Organizational Performance *OrgPerf	Please share your opinion about the performance of the company comparing its aspects to your main competitors:
	1. Overall financial situation of the company
	2. Job performance
	3. Quality of products or services (reliability, diligence)
	4. Innovativeness of products or services
	5. Modernity of applied technological solutions
	6. Efficiency of the organization management
	7. Reliability of business processes
	8. Market share
	9. Customers satisfaction
	10. Employees’ satisfaction
Job Performance *JobPerf	Employees always complete the duties specified in their job description
	Employees always meet all the formal performance requirements of their job
	Employees complete their tasks efficiently
	Employees are always able to overcome obstacles to complete their tasks
	Employees are rarely absent from my work
	Employees avoid absenteeism at work without a legitimate reason
	Employees make few mistakes at work
Motivation *Motiva	Employees feel motivated to work
	Employees are willing and ready to carry out the tasks entrusted to them at the level of a satisfying organization
	Employees are willing and ready to allocate extra effort allowing exceed the requirements posed in front of them
Satisfaction *Satisf	Generally speaking, employees are very happy with their work
	Employees do not often think about resigning from work
Organizational commitment *OrgCom	Employees would be very happy to spend the rest of their career with my organization
	Employees really feel as if organization’s problems are their own
	Employees do feel a strong sense of belonging to organization

Source: own work.

Hiring and Employee Development were described based on research of Aurelia and Momin [47]. The constructs contains 1 item, which is assessed based on a 5 points’ Likert scale [47].

Digitalization was taken from the results of Minbaeva [165]. The construct contains 1 item, which is assessed based on a 5 points’ Likert scale [165].

Job Redesign was measured based on research of Aurelia and Momin [47] and Vardarlıer [54]. The construct contains 2 items, which are assessed based on a 5 points’ Likert scale [47, 54].

COVID-19 Training measurement was build based on concept taken from Hackman and Oldham [166]. The construct contains 1 item, which is assessed based on a 5 points’ Likert scale [166].

Communication was borrowed from Carnevale and Hatak [167]. The construct contains 1 item, which is assessed based on a 5 points’ Likert scale [167].

Wellbeing was developed based on results of Agarwal [168] and Aurelia and Momin [47]. The construct contains 3 items, which are assessed based on a 5 points’ Likert scale [47, 168].

Corporate Social Responsibility was measured based on research Agarwal [168]. The construct contains 1 item, which is assessed based on a 5 points’ Likert scale [168].

Organizational Performance was build based on the Balanced Scorecard concept and the assessment was determined in comparison to the main competitors. The construct contains 10 items, which are assessed based on a 5 points’ Likert scale (from “much worse than competitors” to “much better than competitors” with a middle point: “as competitors”) [169].

Job Performance was described based on the task proficiency, task meticulousness and work discipline. The construct contains 7 items, which are assessed based on a 5 points’ Likert scale [170–172].

Motivation measurement was build based on concept taken from Hackman and Oldham [173]. The construct contains 3 items, which are assessed based on a 5 points’ Likert scale [173].

Satisfaction was determined based on results of Fields [174]. The construct contains 2 items, which are assessed based on a 5 points’ Likert scale [174].

Organizational commitment was measured based on research of Meyer and colleagues [175]. The construct contains 3 items, which are assessed based on a 5 points’ Likert scale [175].

All the above-mentioned 5 points’ Likert scales are described from “strongly disagree” to “strongly agree” with a middle point “neither agree nor disagree”. The exception is Organizational Performance, the measurement of which is included in the description of the construct.

### 4.3 Research results

In order to verify the proposed set of hypotheses, which are constructing a hypothetical model given in Fig 1, the path analysis was used–a statistical reasoning based on structural equation modelling. It should be noted that path analysis is an analytic technique, which “enables to specify the proposed network among factors and then test the adequacy of the proposed network to explain relations among data collected” [176], it is a statistical reasoning method well-suited for the purpose of this study [177].

The path analysis was executed using IBM SPSS AMOS, using bootstrapping technique in order to obtain larger sample for the analysis.

#### 4.2.1 Variables scales analysis

In order to be able to assess whether the discussed scales can be used in the study, Cronbach’s α and Factor Analysis were implemented (Table 3). This approach seems to be sufficient due to the fact that the scales used have been previously validated by the creators [178]. The calculations were not performed for Hiring, Employee Development, Digitalization, COVID-19 training, Communication Corporate Social Responsibility, as they are single—item constructs. The systematic method variance was controlled to ensure no common method bias. The obtained results allow to conclude that almost all measurement scales are well-fitted, reliable and coherent. Moreover, discriminant validity was tested to ensure that latent variables that represent different theoretical concepts are statistically different (all HTMT values were below 0,68, which allows to confirm that the chosen variables may be used for path analysis).

**Table 3:** Reliability of scales.

Variable	Number of scales	Cronbach’s α	Factor Analysis
Hiring	1	-	-
Employee Development	1	-	-
Digitalization	1	-	-
Job Redesign	2	0,552	69,102
COVID-19 training	1	-	-
Communication	1	-	-
Wellbeing	3	0,473	49,227
Corporate Social Responsibility	1	-	-
Organizational Performance	10	0,846	42,087
Job Performance	7	0,812	47,428
Motivation	3	0,639	58,112
Satisfaction	2	0,594	71,132
Organizational commitment	3	0,666	59,983

Source: own work.

#### 4.3.1 Latent variable analysis

As an initial step, the COVID-19 oriented HRM strategies latent variable was verified. Including set of those strategies as a latent variable in the model is fully justified, as any purposefully selected set of strategies will never explain in full the variability of entire strategy phenomenon and random factor should be always accounted for [179]. As shown in Table 4, all indicated strategies are included in the model as factors statistically significantly shaping the latent variable.

**Table 4:** Latent variable verification.

			Estimate	S.E.	C.R.	P
TrainC	<---	HRMS	1,000			
ReDesiC	<---	HRMS	,984	,080	12,296	***
CSRC	<---	HRMS	,862	,090	9,582	***
WellbeC	<---	HRMS	,859	,073	11,693	***
CommuC	<---	HRMS	,962	,092	10,408	***
DigitC	<---	HRMS	,990	,099	9,992	***
HiringC	<---	HRMS	,681	,092	7,411	***
EmpDevC	<---	HRMS	,736	,094	7,826	***

Source: own work.

#### 4.3.2 Path analysis

Based on that, the path analysis was performed. The overview of the obtained model is presented in Table 5 (regression coefficients), Tables 6 and 7 (total and direct effects). The assessment of the model was performed. The fit of the model was measured at first and assessed with CFI (which should be above 0,8) and RMSEA (which should be below 0,2). The obtained model was statistically significant and well-fitted: Chi2 (63) = 173,420; p = 0,005; CFI = 0,942; RMSEA = 0,086. Hence, the conclusions can be formed based on the obtained results.

**Table 5:** Regression Weights for the model.

			Estimate	S.E.	C.R.	P
Motiva	<---	HRMS	,939	,075	12,573	***
Satisf	<---	HRMS	,903	,080	11,251	***
OrgCom	<---	HRMS	,881	,072	12,224	***
JobPer	<---	Motiva	,254	,042	5,989	***
JobPer	<---	Satisf	,138	,037	3,760	***
JobPer	<---	OrgCom	,299	,043	6,914	***
OrgPerf	<---	JobPer	,611	,038	15,903	***

Source: own work.

**Table 6:** Standardized total effects among variables within the model.

	HRMS	OrgCom	Satisf	Motiva	JobPer
OrgCom	,881	,000	,000	,000	,000
Satisf	,903	,000	,000	,000	,000
Motiva	,939	,000	,000	,000	,000
JobPer	,626	,299	,138	,254	,000
OrgPerf	,382	,182	,084	,155	,611

Source: own work.

**Table 7:** Standardized direct effects among variables within the model.

	HRMS	OrgCom	Satisf	Motiva	JobPer
OrgCom	,881	,000	,000	,000	,000
Satisf	,903	,000	,000	,000	,000
Motiva	,939	,000	,000	,000	,000
JobPer	,000	,299	,138	,254	,000
OrgPerf	,000	,000	,000	,000	,611

Source: own work.

The results based on the well-fitted model obtained with path analysis show that all relations within the model are statistically significant (Table 5 showing p value less than 0,01 in all cases). Moreover, the results show that the influence of HR COVID-19 -related strategies is stronger in case of motivation and organizational commitment as mediators than in case of job satisfaction as a mediator in the model. It is confirmed by standardized indirect effects (calculated as total effect (Table 6)–direct effect (Table 7)), which are higher in case of organizational commitment and motivation, than in case of satisfaction. However, in all three cases the indirect effect occurs, confirming that HR COVID-19 -related strategies influence organizational performance through the entire set of variables included in the model.

Therefore, the obtained results of the path analysis confirmed that HR COVID-19 -related strategies influence organizational performance through job-related attitudes (work motivation, job satisfaction and organizational commitment) and job performance, which allows to accept H1-H4 hypotheses.

## 5. Discussion

The article examined the impact of COVID-19 oriented HRM strategies on the job and organizational performance through job-related attitudes. The proposed model of COVID-19—related HRM strategies influence organizational performance through job-related attitudes (work motivation, job satisfaction, and organizational commitment) and statistically significant job performance. As path analysis was used to validate the model, this means that the proposed network of connections between factors explains the relationship between the collected data [172]. The parameters for the constructed structural path model so the degree of fit of the model are in line with literature indications and they are as follow: Chi2 (63) = 173,420; p = 0,005; CFI = 0,942; RMSEA = 0,086, so it proves that the model is well-fitted.

In more detail, it means that a positive relation was established between proposed COVID-19 –related HRM strategies and job-related attitudes. All the HRM strategies identified in this research, forming one latent variable—HRM strategy (each of the HRM strategies maintaining statistical significance). The selected HRM strategies mitigate the negative effects of COVID-19 on job-related attitudes of employees and their job performance and organizational performance. It means that the theory that there is a combination of ’soft’ and ’hard’ COVID-19—related HRM strategies which maximally minimize the negative effects of COVID-19 pandemic in organizations is confirmed. Therefore, due to the changes in the mode of work, it is necessary to include in the HRM strategies taking care of the employees’ satisfaction. Therefore, it seems that it is important is to include the HRM strategies such as job redesign focusing on the hybrid or remote work conditions, and thus also digitalization enabling remote work. Necessarily is also to improve the communication skills from employer to employees especially regular informing about the important aspects of the pandemic to prevent the disorganization feeling. Another aspect is to retain the employees’ wellbeing focusing on keeping social relations between employees remotely, CSR to keep employees feeling safe about their health—called "soft" HR COVID-19—related strategies. Moreover, to ensure an appropriate level of employees’ job performance, it is necessary to support them also in adapting to new working conditions by providing training and development focusing on the acquire the new skills necessary to effectively work in the new condition. On the other hand, from a financial point of view, the best approach during the crisis is to reduce costs, which can guarantee financial stabilization and freezing the money necessary to remain the organization in case of reducing or declining demand [46, 50]. Therefore, the identified COVID-19 –related HRM strategies should also include such practices as withholding hiring, reducing the training and development budget.

Analysis of the following model paths shows that there is a statistically significant effect of the latent COVID-19 –related HRM strategies variable on job-related attitudes, i.e.: work motivation, job satisfaction, and organizational commitment. This means that the HR strategy used in organizations during COVID-19 has a direct, statistically significant impact on employees’ behavior by increasing their job motivation, job satisfaction, and their commitment to the organization. This, in turn, enhances their job performance, passing on to organizational performance. Indeed, there is no direct effect of HRM strategies on organizational performance, or a direct effect is weaker than the impact included the job-related attitudes.

HRM, as a modern approach to personnel management, sees the employee as the organization’s greatest capital. The appropriate choice of HRM strategies and the proper combination of them into a single strategy can be a kind of panacea for motivating employees and influencing their work attitudes and the way they work [132, 133]. Taking actions to ensure the employees’ comfortable at work, including working from home, has an appropriate standard of communication with colleagues, and socializes even at a remote site, will help to reduce the fears caused by the difficult situation such as the COVID-19 pandemic and the necessary changes that followed it. Such actions to care for the employee, their wellbeing, and safety, especially in a difficult situation, will positively affect their perception towards the organization thus increasing employees’ job satisfaction [138]. Moreover, they will give a clear picture to the employees that they are a key resource for the organization. This will strengthen the employee’s sense of trust and confidence towards the employer, which plays a particularly significant role in building employees’ commitment to the organization [151, 152].

Each of these job-related attitudes appropriately stimulated by HRM strategies that aim at mitigating the negative impact of the COVID-19 pandemic will bring measurable benefits to the organization by supporting employees’ job performance [130, 131]. The employees’ positive perception of the job and the organization will have a motivating effect on the employees to put in maximum effort at work so that they can also achieve their personal goals [132, 134, 135]. In the aspiration to satisfy one’s own needs through work, commitment to the organization is also rooted, which by releasing the employees’ positive energy towards work, results in positive behavior of the employees in the workplace. Moreover, it also determines positive behavior regarding the organization by focusing on the realization and achievement of the organization’s goals [155].

Organizational commitment also affects the quality of work, which is closely related to employees’ job performance [156]. The results obtained provide a useful knowledge base for organizations on selecting personnel strategy in the context of the crisis caused by the COVID-19 pandemic. Therefore, it provides information on how, by stimulating job-related attitudes, to achieve an appropriate level of employees’ job performance, which is necessary to achieve an optimum value of organizational performance and to keep the organization’s position on the market. It is clear that during the crisis caused by the COVID-19 pandemic, which forced the numbers of changes inside the organizations, it was necessary also to adjust the HRM strategies. Unfortunately, the organizations focusing on keeping the market position in a difficult time very often forgot about the recognition of which HRM strategies and in which shape should be adapted to the organization to ensure the employees’ safety and their job performance. Thanks to the conducted research, organizations not only get the ready-to-use set of the HRM strategies mitigating the negative impact of COVID-19 on employees. Organizations also obtain knowledge of how the specific HRM strategies should be shaping to keep the employees’ job performance and organizational performance. From the organization’s point of view, this is very important because both: job performance and organizational performance are the key value, which, in times of crisis and uncertainty on the market, could determine the organization’s success.

## 6. Conclusions

The article explains the phenomenon of the COVID-19 oriented HRM strategies’ role in shaping job performance and organizational performance through job-related attitudes in times of crisis. Consider the pandemic caused by a sudden event like COVID-19, this topic is very important and current for organizations. This is because it provides information on how to shape HRM strategy to maintain employees’ job performance, positively influencing organizational performance, thus determining the organization’s continued existence on the market. In more detail, it has been shown that there is a set of HRM strategies that combination can ensure the strengthening of the employees’ job performance and organizational performance through work motivation, job satisfaction, and organizational commitment. Thus, also could have a positive indirect impact on job performance and finally on organizational performance. This set of HRM strategies can be divided into two groups. The first group, called “soft” HRM strategies are focusing on keeping employees’ satisfaction and wellbeing and also preparing employees for the new working conditions and taking care of their health and safety. The second group, called “hard” HRM strategies included such as strategies that have to save money in the organization by withholding hiring and reducing the budget for training and development. The combination of both groups causes that employee not only are comfortable with the new reality, feel that the organization cares about their health and safety. Moreover, due to the rational reduction of costs spent by the organization, they also feel the security of keeping their jobs despite difficult conditions. This sense of security increases the trust of the employer, which effectively maintains a satisfactory level of job performance, directly influencing organizational performance.

The obtained results allowed us to achieve the aim of this article–empirical verification of the indicated COVID-19 oriented HRM strategies in strengthening the job performance and organizational performance through job-related attitudes. Unfortunately, the topic of COVID-19 –related HRM strategies influence job and organizational performance through job-related attitudes is not fully exhausted in the article and has some limitations. First of all, the research was conducted in a limited group of organizations—378 entities were taken into account. Second of all, the chosen organizations were operating in Poland, where the impact of the COVID-19 pandemic on the organizations can be significantly different than in another countries. Third of all, the research was done during 2^nd^ wave of pandemic, and the characteristics of environmental features during upcoming waves have the potential to change. Fourth of all, the method of selecting elements for the sample was purposive, which is related to the limitations of the conclusions. Fifth of all, only selected characteristics of the organization have been taken into account—the size of the organization and the main source of income. Finally, the use of descriptive statistics has some methodological limitations.

It seems that despite the above limitations, the presented research is a solid step, which may be a starting point for further research. Certainly it is worth considering which of the other HRM strategies could also be valid for shaping the job performance and organizational performance in times of COVID-19 crisis. In addition, as it was mentioned, it seems to be necessary to expand research by referring to certain organizations in another country, for which the set of HRM strategies could be different. Also, worth considering is the inclusion of additional job-related attitudes, such as turnover intention or organizational trust. Subsequently, the scope can also be extended to include the issues of leadership, significantly influencing the shape of the organization’s activities, as well as the characteristics of employees. It seems advisable to use a qualitative approach to the studied phenomenon, to increase the number of examined entities and to select a different method of selecting elements for the sample.

Nevertheless, independently of scientific values, this article also has practical ones. It seems very important to define the set of COVID-19—related HRM strategies that have an impact on job performance, and consequently on organizational performance, which is crucial for the operation and survival of the organization. Entrepreneurs were provided with guidelines on how to manage these strategies. The necessity of shaping the employment assurance process was indicated, which should take into account the current and future demand for employees, with particular emphasis on turbulent environmental conditions. Methods of approaching employee development have been defined, especially when it may be necessary to reduce financial outlays for training. As a consequence of the spread of contamination is the need for social isolation, solutions concerning the transfer to remote work were proposed. The issues of digitalization, which is inseparable from working from home, were discussed, and a change in work processes was proposed to adapt to the new reality (job redesign). The above changes could not be introduced without adjusting communication in the organization to the needs caused by the COVID-19 pandemic. Finally, in order to avoid negative consequences in the emotional sphere of employees, the importance of ensuring wellbeing of stakeholders in and outside of the organization was emphasized. Therefore, the issues of wellbeing of employees were discussed, as well as corporate social responsibility, which has a broader scope. All the above-mentioned issues are the starting point for practical actions aimed at improving the operation of the organization during the coronavirus threat. It is also worth emphasizing that specific HRM strategies, which have a significant impact on job performance and organizational performance through the job-related attitudes: work motivation, job satisfaction, and organizational commitment is valid besides the organization’s size and main source of income.

In summary, the COVID-19 pandemic is a phenomenon that has broken the previously known and relatively stabilized order in the management of contemporary organizations. The observable effects of the coronavirus outbreak have significantly influenced the approach to management, including Human Resource Management. The long-term consequences of these changes are neither known nor foreseeable. However, it seems necessary to diagnose the current situation and take into account the possibility of using the experience so far. Due to the predictions it will not be an immediate end of the pandemic, and also the indicate a high probability of similar phenomena in the future.

## Supporting information

S1 File (ZIP)
